# Early gain in pain reduction and hip function, but more complications following the direct anterior minimally invasive approach for total hip arthroplasty: a randomized trial of 100 patients with 5 years of follow up

**DOI:** 10.1080/17453674.2018.1504505

**Published:** 2018-10-23

**Authors:** B Harald Brismar, Ola Hallert, Anna Tedhamre, J Urban Lindgren

**Affiliations:** a Department of Clinical Sciences, Intervention and Technology, Karolinska Intitutet, Department of Orthopedics, Karolinska University Hospital, Stockholm;;; b Department of Orthopedics, Södertälje Hospital, Södertälje;;; c Department of Orthopedics, St Göran Hospital, Stockholm, Sweden

## Abstract

Background and purpose — The minimally invasive direct anterior (DA) approach for total hip arthroplasty (THA) is supposed to reduce surgical tissue trauma. We hypothesized that patients operated with the DA technique would have less postoperative pain and better hip function compared with a group operated with a conventional direct lateral (DL) approach.

Patients and methods — 100 patients with hip osteoarthritis scheduled for THA were equally randomized to surgery through either DA or DL. Pain was assessed on a VAS scale, hip function with TUG, 10mWT, HHS, and quality of life with EQ-5D. Patients were followed up after the first 3 days, 8 weeks, and at 1 and 5 years postoperatively.

Results — The DA group registered less pain with activity on the second day (VAS 42 vs. 55), performed TUG 6 seconds faster on the third day and had 8 points higher HHS and higher EQ-5D index (0.86 vs 0.78) at 8 weeks; all differences were statistically significant. No clinically relevant differences between groups in pain, hip function, or quality of life were seen at 1 or 5 years. 7 surgical approach related complications appeared in the DA group, none in the DL.

Interpretation — The results indicate that the presumably less traumatic approach results in reduced immediate postoperative pain and better hip function and higher quality of life in the early postoperative period. However, this positive effect is not seen at later time points. Instead, complications appear to be over-represented, thus questioning the use of the method.

A continuous ambition towards further improvement of functional outcome and shorter hospital stay has led to techniques aiming at reducing surgical tissue trauma in total hip arthroplasty (THA). The minimally invasive direct anterior (DA) single incision approach uses an internervous plane without detachment of tendons or splitting of muscles. A number of observational cohort studies have been published describing the technique (Kennon et al. 2003, Siguier et al. 2004, Matta et al. 2005). Several reports indicate less pain in the immediate postoperative period following DA-THA compared with other approaches (Goebel et al. 2012, Barrett et al. 2013, Mjaaland et al. 2015) and higher quality of life measures up to 1 year postoperatively were found in 1 study (Restrepo et al. 2010).

Potential risks associated with minimally invasive surgical techniques are malpositioning of implants and unintended tissue damage due to a reduced surgical field. A higher complication rate for minimally invasive hip arthroplasty has been reported from the Swedish hip arthroplasty registry (Hailer et al. 2012) and also in a study focusing on DA-THA (Spaans et al. 2012). However, others have reported excellent results using the DA technique (Siguier et al. 2004, Restrepo et al. 2010). MRI studies following DA-THA have shown less gluteus muscle fatty atrophy at 6–12 months postoperatively (Bremer et al. 2011, De Anta-Díaz et al. 2016) compared with DL-THA. Also, a cadaveric dissection study implies less gluteus medius damage following DA (van Oldenrijk et al. 2010).

We compared the outcome following DA-THA with a standard direct lateral approach (DL) for THA focusing on pain, hip function, quality of life, and complications. Our primary hypothesis was that a surgical technique not involving intentional detachment of muscle insertions (the DA technique) would lead to less pain in the immediate postoperative period. Our secondary hypothesis was that hip function and quality of life would be better following DA-THA. No difference in complications was expected. At the start of this study, no large randomized trial on this subject had been published.

## Patients and methods

This was a single-center randomized parallel group study with equal randomization to 2 groups. The study was conducted at the orthopedic department, Karolinska University Hospital, Huddinge, Sweden. Patients with hip osteoarthritis referred for hip arthroplasty were, after consent, informed and asked for participation in the study. Exclusion criteria were dementia, neuromuscular disorders, alcohol/drug abuse, and previous hip surgery on the affected side. 100 consecutive patients were randomly allocated by a computer program to either surgery through a direct anterior (DA) single incision approach (Rachbauer 2005) or a direct lateral (DL) transgluteal approach (Duparc et al. 1997), inclusion period November 2006 to April 2008. There were 50 patients in each group. Patient preoperative characteristics were similar between the 2 groups (Table 1). Each patient was given a consecutively numbered sealed envelope containing an allocation paper put into the envelope by an independent research manager not further involved in the study. The surgeon and patient were blinded prior to the opening of the envelope.

**Table 1.** Characteristics of patients who had a total hip arthroplasty through a direct anterior (DA) approach or a direct lateral (DL) approach. Values are median and inter-quartile range or proportion of patients

	DA	DL
Patient characteristics	n = 50	n = 50
Sex (F/M)	32/18	33/17
Age (years)	66 (58–74)	67 (60–76)
Smoker (yes/no)	6/42	6/42
Weight (kg)	80 (68–90)	76 (64–86)
Height (cm)	168 (164–175)	167 (162–175)
Body mass index	27 (24–29)	27 (24–30)
ASA grade (1/2/3)	12/30/8	10/35/5
1 hip OA/2 hip OA/2 hip THA	26/17/7	25/20/5
Side (R/L)	30/20	30/20

Briefly, the DA was carried out with the patient supine on a standard operating table allowing angulation at the level of the hip. The skin was incised at a point 2 fingerbreadths lateral to the anterior sciatic spine and extended 8–10 cm distally. The tensor fascia lata and gluteus medius muscles were retracted laterally and the sartorius and rectus muscles medially exposing the capsule. A special offset acetabular reamer and an offset broach handle were used. The DL was performed with the patient in a lateral decubitus position. Access to the joint was gained through a 10–20 cm long skin incision centered over the greater trochanter, splitting the fascia lata/gluteus maximus and detachment of the caudal 2/3 of the gluteus medius and the entire gluteus minimus tendon insertions. Finally, the capsule was excised anteriorly. The muscle tendons were reattached to the trochanter by osteosutures following implantation.

All patients had uncemented implants (Accolade stem and Trident PSL cup, Stryker, Kalamazoo, MI, USA). 92 patients received spinal anesthesia (47 DA and 45 DL) and 8 general anesthesia (3 DA and 5 DL). 2 surgeons performed all procedures. Both surgeons were well acquainted with both techniques having performed at least 40 independent procedures with the DA technique and using the DL technique for several years before the onset of the study.

Time for surgery was from skin incision to skin closure. The anesthesia nurse estimated perioperative blood loss from suction and surgical swabs. Blood samples were drawn preoperatively, and at 6 hours and 2 days postoperatively; C-reactive protein (CRP), interleukin-6 (IL-6), and hemoglobin levels were analyzed.

All patients were treated postoperatively according to the same pain management protocol including a regular long-acting morphine analog the first day (oxycodone 10 mg 2 times daily), regular paracetamol (1 g 4 times daily) and short-duration morphine (oxycodone or morphine) on demand. The long-acting dose was adjusted with regard to the previous day’s morphine consumption. The total sum of equipotent doses of oral morphine consumed 3 days postoperatively was estimated (10 mg oral oxycodone =20 mg oral morphine, 10 mg iv morphine =30 mg oral morphine). Patients were asked to keep track of how many days after discharge from hospital they continued to use morphine.

Pain in the operated hip at rest and during activity was estimated by the patients on a VAS scale. VAS was recorded preoperatively, on the morning of day 1 and day 2, at 8 weeks, 1 year, and 5 years postoperatively. Hospital stay was defined as the number of postoperative nights in the hospital and in those cases where rehabilitation was needed an additional 3 days were added as a default. No patient was sent to a rehabilitation facility earlier than the fourth postoperative day. All patients were given the same postoperative physiotherapy regime focusing on independent rising from bed and walking with aids. Discharge criteria were: ability to walk 20 meters independently and adequate pain management with oral analgesics.

Timed up and go (TUG) (Kennedy et al. 2002) and 10-meter walk test (10mWT) (Salbach et al. 2001) were performed preoperatively, on day 3, and at 8 weeks, 1 year, and 5 years postoperatively. EQ-5D and Harris Hip Score were assessed at 8 weeks, 1 year, and 5 years postoperatively.

Patients or their caretakers were asked at 8 weeks, 1 year, and 5 years if any complication had occurred related to the hip surgery. 99 patients had 8 weeks’ follow-up (1 patient, DL group, missed the 8-week follow-up). One patient had died at 1 year (DA group) and 99 came to follow-up. At 5 years 87 patients had complete follow-up (DA 45 and DL 42). 3 patients in each group had died, 1 had been revised (DA group), 1 declined further follow-up (DL) and 5 patients were unable to respond to questions due to dementia or frailty (DA 1 and DL 4). The 13 patients who did not come to follow-up were all checked with a phone call and/or medical chart investigation.

### Statistics

Categorical data were summarized using frequency counts or percentages and continuous data were presented as means and 95% confidence intervals or as medians and inter-quartile ranges (Q1–Q3). A linear mixed model, which allows for missing data, was used to analyze repeated measurements of VAS, EQ5-D, 10mWT, TUG, and Harris Hip Score (HHS) taken during the hospital stay and at follow up until 1 year. The fixed factors in the model were Group (DA and DL), Time (2, 3 or 4 time points) and the Group x Time interaction. In case of a significant interaction, simple main effects tests were performed, i.e., effects of 1 factor holding the levels of the other factor fixed. We used the default method ddfm = between in SAS in order to compute the degrees of freedom. The most appropriate covariance structure for our outcome measures was unstructured and heterogeneous compound symmetry. The variables TUG and 10mWT were ln-transformed prior to these analyses to compensate for their positively skewed distribution. Student’s t-test for independent samples and the Mann–Whitney U test were used in analyzing data at 1 time point. Fisher’s exact test was used to analyze categorical data. A p-value <0.05 was considered statistically significant.

A sample size of 100 patients was chosen in order to get statistical power of at least 80%, assuming a standard deviation of 20 for VAS registration. The primary endpoint was pain in the first postoperative days. The minimal clinically important difference was set to 13 for VAS. 5-year results were analyzed separately since the number of patients under study had diminished considerably and unequally between groups.

We used Statistica 10.0 (www.statsoft.com), StatSoft (www.statsoft.com), and SAS System 9.1 (SAS Institute, Cary, NC, USA) for the analyses.

### Ethics, funding, and potential conflicts of interest

The Central Ethical Review Board, Stockholm approved the protocol (2005/1508-31) and informed consent was obtained from all participants. Stryker unconditionally sponsored the study. The authors declare no conflicts of interest.

## Results

All patients were operated according to their allocated method and there were no intraoperative complications. Hospital stay was 1 day shorter in the DA group (Table 2) and only 1 patient had to be sent to a rehabilitation facility compared with 5 in the DL group.

**Table 2.** Surgical and early postoperative outcome parameters for patients who had a total hip arthroplasty through a direct anterior (DA) approach or a direct lateral (DL) approach

	DA	DL	
Outcome parameters	n = 50	n = 50	p-value
Operation time (min)**^a^**	101 (87–112)	80 (71–86)	< 0.001 **^c^**
Intraoperative blood loss (mL) **^a^**	325 (200–500)	300 (250–450)	0.7**^c^**
IL-6, 6 h postoperatively (pg/mL)**^b^**	56 (46–67)	77 (60–94)	0.05**^d^**
CRP 2 days postoperatively (mg/mL)**^b^**	211 (191–231)	238 (219–257)	0.06**^d^**
Morphine consumption (mg) 3 days**^a^**	140 (110–197)	175 (140–234)	0.02 **^c^**
Postoperative days on morphine**^a^**	12 (6–17)	14 (8–18)	0.05 **^c^**
Postoperative hospital nights**^a^**	3 (3–4)	4 (3–5)	0.006 **^c^**

**^a^**Median and inter-quartile range.

**^b^**Mean and 95% confidence intervals.

**^c^**Mann–Whitney U test,

**^d^** T-test.

**Figure 1.**
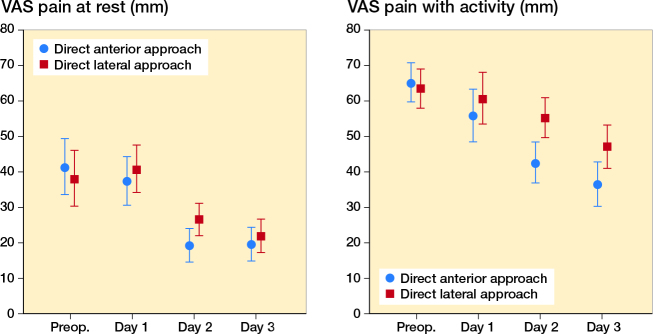
VAS for patients who had a total hip arthroplasty through a direct anterior (DA) approach or a direct lateral (DL) approach preoperatively and at 3 time points postoperatively. The DA group had lower VAS pain at rest 2 days (p = 0.03) and lower VAS pain with activity 2 days and 3 days postoperatively than the DL group (p = 0.002 and p = 0.02). Between- and within-group comparisons were performed using a linear mixed model. Circles indicate DA means, boxes indicate DL means, error bars indicate the 95% confidence intervals of the DA and DL means.

Patients in the DA group registered less pain during activity and at rest on the second day postoperatively and also the third day during activity. There was no clinically relevant difference at any other time point (Figure 1). 13 patients in the DA group did not require any extra, oral or parenteral, morphine in the first 3 postoperative days compared with 5 patients in the DL group (p = 0.07). The DA group had a 20% lower morphine analgesic consumption measured as the total amount of morphine consumed by the third day and quit oral morphine analgesics 2 days earlier than the DL group (Table 2). There was no important difference between groups regarding pain at rest or during activity at 8 weeks, 1 year, and 5 years postoperatively (Table 3).

**Table 3.** Pain in the operated hip at rest and during activity (VAS) at 3 time points postoperatively following total hip arthroplasty through a direct anterior (DA) approach or a direct lateral (DL) approach. Values are absolute number of patients at each time period with VAS =0, VAS 1–30 and VAS >30

	8 weeks		1 year		5 years	
	DA	DL	DA	DL	DA	DL
	n = 50	n = 49	n = 49	n = 50	n = 45	n = 42
VAS at rest						
0	36	31	45	41	38	38
1–30	11	15	4	6	6	3
> 30	3	3	0	3	1	1
VAS during activity						
0	26	21	44	41	36	33
1–30	21	22	4	6	7	7
> 30	3	6	1	3	2	2

In the DA group mean Il-6 was 27% lower at 6 hours postoperatively and mean CRP was 11% lower 2 days postoperatively (Table 2).

**Figure 2.**
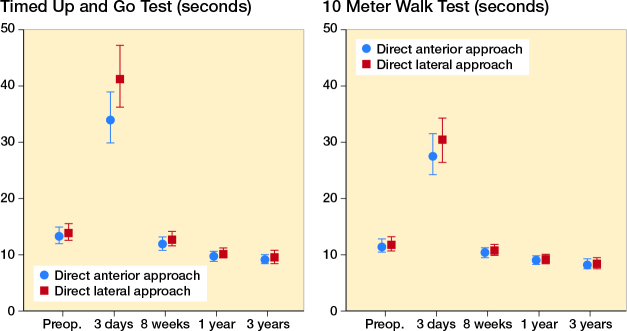
Timed up and go (TUG) and 10-meter walk test (10mWT) for patients who had a total hip arthroplasty through a direct anterior (DA) approach or a direct lateral (DL) approach preoperatively and at 4 time points postoperatively. The DA group performed TUG faster than the DL group 3 days postoperatively (p = 0.04). Between- and within-group comparisons were performed using a linear mixed model. Circles indicate DA means, boxes indicate DL means, error bars indicate the 95% confidence intervals of the DA and DL means.

TUG and 10mWT deteriorated from preoperative values to 3 days postoperatively but already at 8 weeks postoperatively they had improved and continued to improve until 1 year. The DA group performed TUG 6 seconds faster than the DL group at 3 days postoperatively. There were no clinically relevant differences between groups at any other time point (Figure 2).

**Figure 3.**
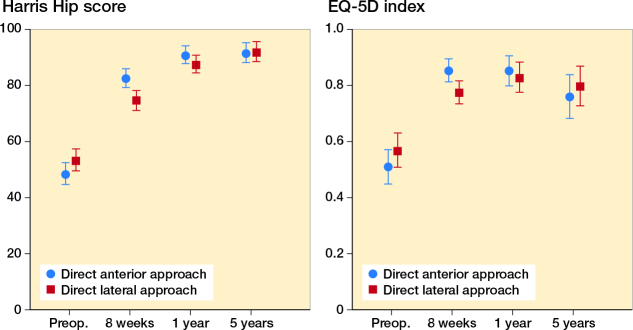
Harris hips score (HHS) and EQ-5D index for patients who had a total hip arthroplasty through a direct anterior (DA) approach or a direct lateral (DL) approach preoperatively and at 3 time points postoperatively. The DA group had lower HHS and EQ-5D 8 weeks postoperatively than the DL group (p = 0.002 and p = 0.009). Between- and within-group comparisons were performed using a linear mixed model. Circles indicate DA means, boxes indicate DL means, error bars indicate the 95% confidence intervals of the DA and DL means.

Both groups improved in HHS and EQ-5D from preoperative levels to 8 weeks and 1 year postoperatively. At 8 weeks the DA group had 8 points higher HHS and 0.08 higher EQ5-D means than the DL group. At 1- and 5-year follow-ups no clinically relevant differences were seen between groups (Figure 3).

At 5-year follow up 7 surgical approach related complications had appeared in the DA group, but none in the DL group (p = 0.01). In the DA group 2 early dislocations were handled by closed reduction, neither of them recurred; 1 early deep infection was resolved following open irrigation, retention of the prosthesis, and antibiotics for 6 months; 1 patient dislocated 22 months postoperatively and was revised at 2 years and 9 months due to a pseudotumor; 1 patient developed hip pain 4 years postoperatively and was diagnosed with an iliopsoas cyst, revision was planned at 5 years; 1 patient had a late dislocation at 4 years and 7 months postoperatively and was handled by closed reduction, and, finally, 1 patient developed instability–subluxations at 1.5 years postoperatively, but did not find the disability severe enough to motivate revision surgery. Postoperative radiographs were analyzed in all patients with instability and all but one had cups positioned within Lewinnek’s safe zone (Lewinnek et al. 1978). That patient had a cup with 54 degrees of inclination and 16 degrees anteversion; he had 1 dislocation and no later recurrent instability problem. In the DL group, 1 patient developed hyperesthesia from the femoral cutaneous nerve of the opposite, unoperated leg, probably originating from pressure from the table support during surgery. 1 deep venous thrombosis occurred within 3 months in each group. 1 patient in each group had disturbed wound healing. Both healed uneventfully following treatment with oral antibiotics.

## Discussion

In this randomized trial, we found an initial beneficial effect on postoperative pain and hip function, probably due to reduced tissue trauma. However, at later follow-up, that effect disappeared. An unexpectedly high number of complications occurred in the DA group, presumably related to a more demanding surgical technique.

### Pain

Patients had less pain following the DA procedure in the immediate postoperative period. Even though the DA group consumed less total morphine in the first 3 days postoperatively, pain at rest was the same between the 2 groups and notably pain during activity was lower in the DA group. Patients in the DA group also had a shorter postoperative period on opiate analgesics and a shorter hospital stay. It is, therefore, reasonable to believe that the presumably less traumatic minimal invasive technique actually caused less pain. The result is in line with previous reports (Goebel et al. 2012, Mjaaland et al. 2015). Not surprisingly, the pain did not differ between groups after the immediate postoperative period, in concordance with other studies (Restrepo et al. 2010, Reichert et al. 2015).

### Surgical trauma

A technique not involving tendon or muscular division is theoretically attractive, although one has to bear in mind the risk of unintentional muscular trauma due to either excessive traction of tissues or accessory releases in order to gain surgical access. In an attempt to objectively estimate the surgical trauma we measured Il6 and CRP, inflammatory markers reported to be elevated proportionally to the amount of trauma (Watt et al. 2015). Following THA Il-6 has been found to peak rapidly 6 hours postoperatively, while CRP rises more slowly with a peak 2 days postoperatively (Wirtz et al. 2000). The 27% lower Il-6 levels in the DA group could reflect reduced surgical trauma, while the 11% lower CRP levels are more difficult to interpret. Both values varied considerably between individuals within groups and have in a recent study been reported to correlate badly to the postoperative functional outcome (Poehling-Monaghan et al. 2017).

### Hip function

Mean TUG was 6 seconds shorter in the DA group at 3 days and mean HHS 8 points higher at 8 weeks indicating early better hip function. Since there were no clinically important differences between groups at later time points, it seems that the presumed lesser muscular trauma following the DA procedure did not substantially affect hip function in the long run. This is in line with previous reports (Mayr et al. 2009, Restrepo et al. 2010, Parvizi et al. 2016).

### Quality of life

The long-term benefit of reduced surgical trauma and less initial postoperative pain is unclear. In the immediate rehabilitation period, pain and function of the operated joint will certainly affect measures of quality of life such as EQ-5D, illustrated by the mean 0.08 higher EQ-5D at 8 weeks in the DA group. Later, at 1- and 5-year follow up, potential differences in hip function must be quite substantial compared with other factors not related to the previous hip surgery in order to have an impact on daily life.

### Complications

7 of 50 patients in the DA group had an approach-related complication within 5 years from surgery. 4 were directly related to instability. Malposition of the cups did not seem to explain the dislocations. One may speculate that excessive mobilization of the femur in order to be able to insert the femoral implant during a DA procedure could negatively affect posterior stabilizing structures and thereby hip stability. There have been previous reports on high complication rates following MIS-THA procedures (Spaans et al. 2012, Hailer et al. 2012, Müller et al. 2014) and longer learning curves than expected (Spaans et al. 2012) indicating that MIS-THA can be demanding. The higher complication rate in the DA group could represent a still ongoing learning curve. There are reports of more favorable results (Kennon et al. 2003, Siguier et al. 2004) suggesting that a high surgical volume and meticulous improvement in surgical technique probably decrease rates of complications, but in view of our results one may question whether the method is too demanding to master in relation to the potential advantages.

### Limitations

Although this was a randomized trial the result may have been affected by bias since patients and caregivers could not be blinded as the location of the scar revealed the approach. At the time of the study, the method was not widespread, possibly reducing the risk of different expectations linked to either method. Patients were treated by the same protocol and the results were recorded by a physiotherapist not involved in the recruitment of patients. It is possible that the functional tests used were not demanding enough to detect differences between groups at later time points.

### Conclusion

In our hands, the positive early effects of the minimally invasive direct anterior technique do not justify further use of the technique unless complications can be reduced to a similar rate seen using the conventional direct lateral technique. The hypothetical advantage of having undamaged tissue around the hip following a minimally invasive direct anterior procedure could not be proven in our study.

Design of the study, recruitment of patients, and performance of the operations were done by HB, OH, and UL. Follow up by HB and AT. The data was analyzed by HB and UL. The paper was written by HB and reviewed by OH, AT and UL.  

The authors would like to thank Elisabeth Berg, LIME, KI for statistical support.

### Acknowledgement

*Acta* thanks Stefan Bolder and Lars Nordsletten for help with peer review of this study.
